# Successful treatment of refractory ascites in a patient with liver cirrhosis combined with hepatic artery-portal vein malformation: A case report

**DOI:** 10.1097/MD.0000000000036886

**Published:** 2024-01-26

**Authors:** Zhenyu Ge, Kai Wang, Zhaomei Zhang, Xiaoqian Zhang, Peng Sun, Ning Chen, Yang Tan, Tingting Shen, Hongsheng Dai, Wenwen Li

**Affiliations:** a Department Gastroenterology, Affiliated Hospital of Weifang Medical University, Weifang City, Shandong Province, China; b Department Vascular intervention, Affiliated Hospital of Weifang Medical University, Weifang City, Shandong Province, China; c Department Gastroenterology, Affiliated Hospital of Weifang Medical University, Weifang City, Shandong Province, China; d Department Gastrointestinal Surgery, Affiliated Hospital of Weifang Medical University, Weifang City, Shandong Province, China.

**Keywords:** arterio-portal shunt, arteriovenous fistula, artery-portal vein malformation, case report, liver cirrhosis, refractory ascites

## Abstract

**Introduction:**

Hepatic artery-portal vein malformation is rarely encountered in clinical practice. Here, we reported a case of liver cirrhosis combined with hepatic artery-portal vein malformation with refractory ascites as the main symptom. And it was successfully treated by us. The present case demonstrates the role of hepatic artery-portal vein malformation in cirrhotic ascites and the importance of early diagnosis and interventional treatment. This article may provides some experience for the treatment of such patients.

**Patient concerns:**

The patient was a 72-year-old woman with a 40-year history of Hepatitis B virus surface antigen positivity who sought medical advice with a chief complaint of abdominal distension for 1 week.

**Diagnoses:**

Enhanced abdominal computed tomography imaging of this patient revealed liver cirrhosis, splenomegaly, esophageal and gastric varices, massive ascites, and a low-density area in the S4 segment of the liver with an ambiguous boundary. Widening of the left branch of the portal vein was evident, and the portal vein was highlighted in the arterial phase and the venous phase. Digital subtraction angiography revealed substantial thickening of the left hepatic artery, and the administered contrast agent drained through the malformed vascular mass to the thickened left portal vein. Liver cirrhosis combined with hepatic artery-portal vein malformation were diagnosed. And we considered that the artery-portal vein malformation in this patient might be caused by cirrhosis.

**Interventions:**

The patient was applied diuretics, entecavir and transcatheter embolization.

**Outcomes:**

The patient ascites did not resolve significantly when treated with diuretics alone. After the transcatheter embolization, the patient ascites relieved remarkably.

**Conclusion:**

The patient underwent transcatheter embolization for hepatic artery-portal vein malformation, after which her ascites resolved with good short-term curative efficacy. So, the patients who suffered from liver cirrhosis combined with hepatic artery-portal vein malformation and refractory ascites, should be active on transcatheter embolization.

## 1. Introduction

Arteriovenous fistulae (AVFs) can develop as a result of the formation of abnormal short channels that link veins and arteries without passage through the capillary network. Hepatic AVFs present as malformed vascular masses resulting from the aberrant anastomosis of hepatic arteries and hepatic veins or portal veins. AVFs incidence rates in the clinic are relatively low, but a growing number of studies have focused on these malformations in recent years owing to advances in diagnostic imaging techniques such as digital subtraction angiography. Clinical research has demonstrated that hepatic AVFs can significantly influence the efficacy of treatment efforts for hepatocellular carcinoma (HCC) and other hepatic diseases. These AVFs can include hepatic arterial-portal fistulae, hepatic arterial-hepatic venous fistulae, and combined hepatic AVFs. Liver AVFs incidence in patients with liver disease (primarily HCC and liver cirrhosis) is estimated to be approximately 4.3%.^[1]^ Hepatic AVFs formation may be idiopathic or secondary, with the latter accounting for 75% of all cases,^[2]^ with these malformations being secondary to liver diseases such as HCC or cirrhosis, or to various forms of trauma, infection, puncture biopsy, or surgery.

## 2. Case report

### 2.1. Patient information

A 72-year-old woman was admitted to our hospital with a 40-year history of Hepatitis B virus surface antigen positivity complaining of abdominal distension for 1 week. She had never undergone antiviral therapy. One day prior to presentation she had used non-steroidal anti-inflammatory drugs and other analgesics. But she did not used diuretics. She also had a 6-month history of erosive and atrophic gastritis, a 3-year history of lumbar and cervical disc herniation, and an 18-year history of diabetes. She had no history of allergies, alcohol intake, fine needle liver biopsy, interventional radiology, blunt or penetrating liver trauma, hepatectomy, liver cancer, or related diseases. Her father had died of HCC.

### 2.2. Clinical findings

Physical examination revealed a blood pressure of 132/68 mm Hg, a heart rate of 102 bpm, a respiratory rate of 16 breaths/min, and a body temperature of 36.3°C. Examination results revealed palmar erythema, abdominal distension, high abdominal wall tension and shifting dullness positive, but she was otherwise negative for asterixis and jaundice or edema of the lower extremities.

**Figure 1.**
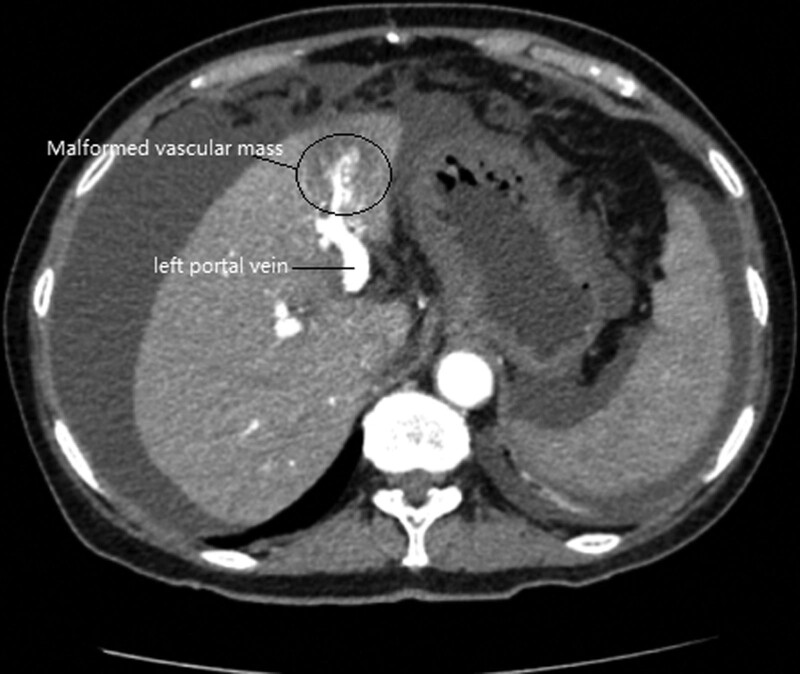
Contrast-enhanced CT scan: An area of abnormal low-density changes with an ambiguous boundary was evident in the S4 segment of the liver. The portal vein was highlighted in the arterial phase, and widening of the left branch of the portal vein was evident. CT = computed tomography.

Following admission, the patient underwent a range of exams and testing, with pertinent test results including the following: Glutamyl transpeptidase 32 U/L, Alkaline phosphatase 62 U/L, aspartate aminotransferase 35 U/L, alanine transaminase 40 U/L, albumin 38.9 g/L, total bilirubin 12.1 µmol/L, conjugated bilirubin 5.0 µmol/L, glucose 15.02 mmol/L, International Normalized Ratio 1.54, Prothrombin Time% 55%, d-dimer 1061 ng/mL, hemoglobin 109 g/L, white blood cell 6.98*10^9/L, platelet 104*10^9/L. Hepatitis B virus surface antigen (+), Hepatitis B virus e antibody (+), Hepatitis B virus core antibody (+), Hepatitis B virus surface antibody (−), Hepatitis B virus e antigen (−), Hepatitis B virus deoxyribonucleic acid quantification 2.33*10^3 IU/mL, Glucoprotein antigen 199 42.8 U/mL, Carbohydrate antigen 125 309 U/mL, Neuron-specific-enolase 37.8 ng/mL. Tests of ascites fluid revealed that it was clear with a water-like appearance, a pH of 9.00, a specific gravity of 1.008, negative qualitative protein results, a red blood cell count of 2000/µL, and a white blood cell count of 174/µL (92% mononuclear, 8% multi-nuclear). Ascites-specific biochemical test results included: total protein 7.6 g/L, albumin 4.5 g/L, glucose 9.8 mmol/L, lactate dehydrogenase 34 U/L, and adenosine deaminase 1.5 U/L. *Mycobacterium tuberculosis* and *M tuberculosis* rifampicin resistance gene detection results were both negative. Additional testing revealed that the patient was negative for markers of autoimmune disease and exhibited normal thyroid and renal function. Chest X-ray revealed a slight increase in bilateral lung texture, calcification of the aortic arch wall, and limited bilateral pleural effusion. Abdominal pelvic plain computed tomography (CT) scan: Slightly low density focus in the S4 segment of the liver. The nature is undetermined. Please conduct further clinical examination. Cirrhosis. Portal hypertension. Splenomegaly. Esophageal and fundus varices. Massive abdominal and pelvic effusion. Nodular thickening of the omentum and local peritoneum, should be identified as possible due to metastasis or exudation. Edema and thickening of the wall of part of the colon. Thickening of the wall of the gallbladder. Reflux disorders or inflammatory changes need to be identified, which should be combined with clinical follow-up. Small hepatic cysts. Please combine clinical. Contrast-enhanced CT imaging similarly revealed: liver cirrhosis, splenomegaly, esophageal and gastric varices, massive ascites, and a 3.9 * 3.3 cm area of low-density changes with an ambiguous boundary located in the S4 segment of the liver. The left branch of the portal vein was widened, and the portal vein was highlighted in the arterial phase and maintained into the venous face, consistent with the presence of a hepatic arterioportal malformation (Fig. 1).

### 2.3. Diagnostic assessment

The patient autoimmune disease index was negative, so the possibility of ascites caused by rheumatic immune disease was ruled out. According to the patient negative tuberculosis indicators, the possibility of tuberculous ascites was ruled out. The patient had normal renal and thyroid function, and the possibility of ascites caused by kidney disease and thyroid disease was ruled out. Enhanced CT of the patient liver showed no signs of tumor, and liver cancer was ruled out.

So based on the patient history, laboratory findings, and imaging findings the patient was diagnosed as: Arteriovenous malformation (left lobe of liver, formation of hepatic arterio-portal vein fistula). Decompensated liver cirrhosis (Child-pugh B). Portal hypertension. Ascites. Esophageal and gastric varices. Splenomegaly. Chronic viral hepatitis B. Active hepatitis. Diabetes. Chronic atrophic gastritis. Iron deficiency anemia. Liver cyst. Lumbar disc herniation. Herniated cervical disc.

### 2.4. Therapeutic intervention

The patient underwent initial treatment with diuretics (Spironolactone 20 mg po tid, Furosemide 20 mg by Micro infusion pump 0.33 mg/min qd) and entecavir (0.5 mg po qn), but diuretic efficacy was poor and she did not experience any significant weight loss.

**Figure 2.**
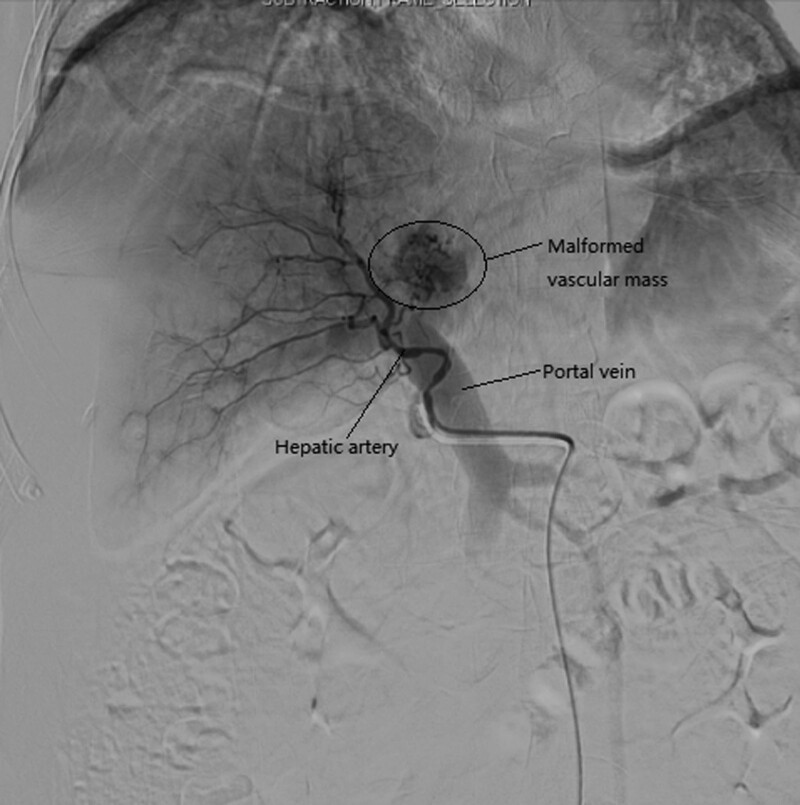
Pre-embolization angiographic image.

**Figure 3.**
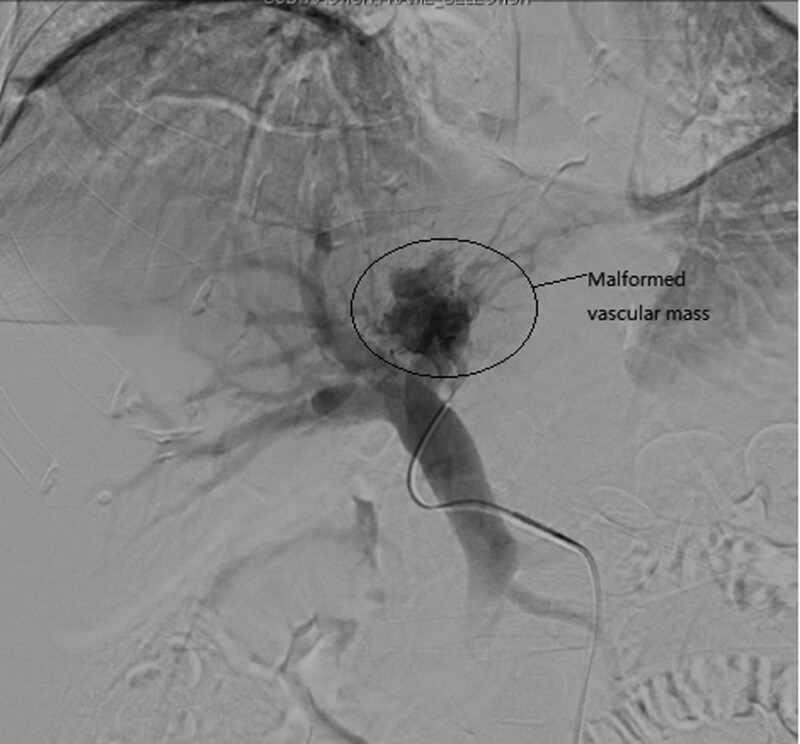
Ultra-selective contrast image.

**Figure 4.**
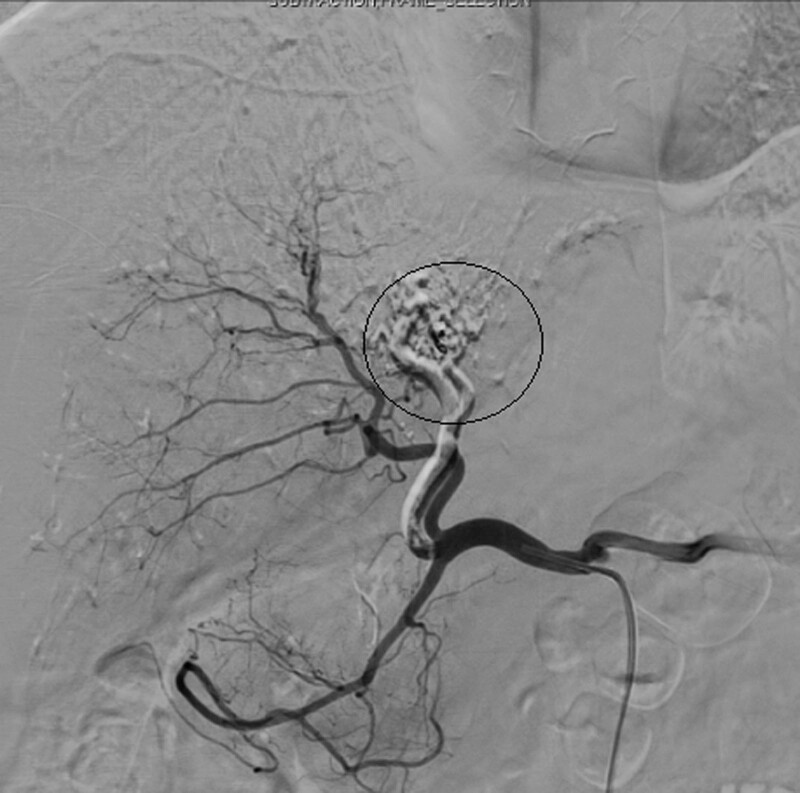
The abnormal vascular mass did not recur following embolization.

Interventional radiologists were consulted about potential embolization and agreed to conduct interventional treatment. Hepatic angiography revealed significant thickening of the left hepatic artery, and contrast agent drained through the malformed vascular mass to the thickened left portal vein. The distribution of the right hepatic artery and its branches was largely normal, although 1 distal branch communicated with the malformed left hepatic vascular mass (Fig. 2). Ultra-selective catheterization of the left hepatic artery was conducted with a microcatheter followed by the injection of a mixture of iodide oil and medical glue (α-butylcyanoacrylate) until the main hepatic artery was fully embolized (Fig. 3). Angiographic reexamination revealed the interruption of blood flow in the left hepatic artery, whereas normal blood flow was evident in the right hepatic artery and its branches (Fig. 4). Arteriovenous malformation of the left lobe of the liver and the formation of a hepatic arterio-portal venous fistula were considered. Transcatheter ultra-selective embolization was successfully performed to treat this hepatic arteriovenous malformation.

### 2.5. Follow-up and outcomes

**Figure 5.**
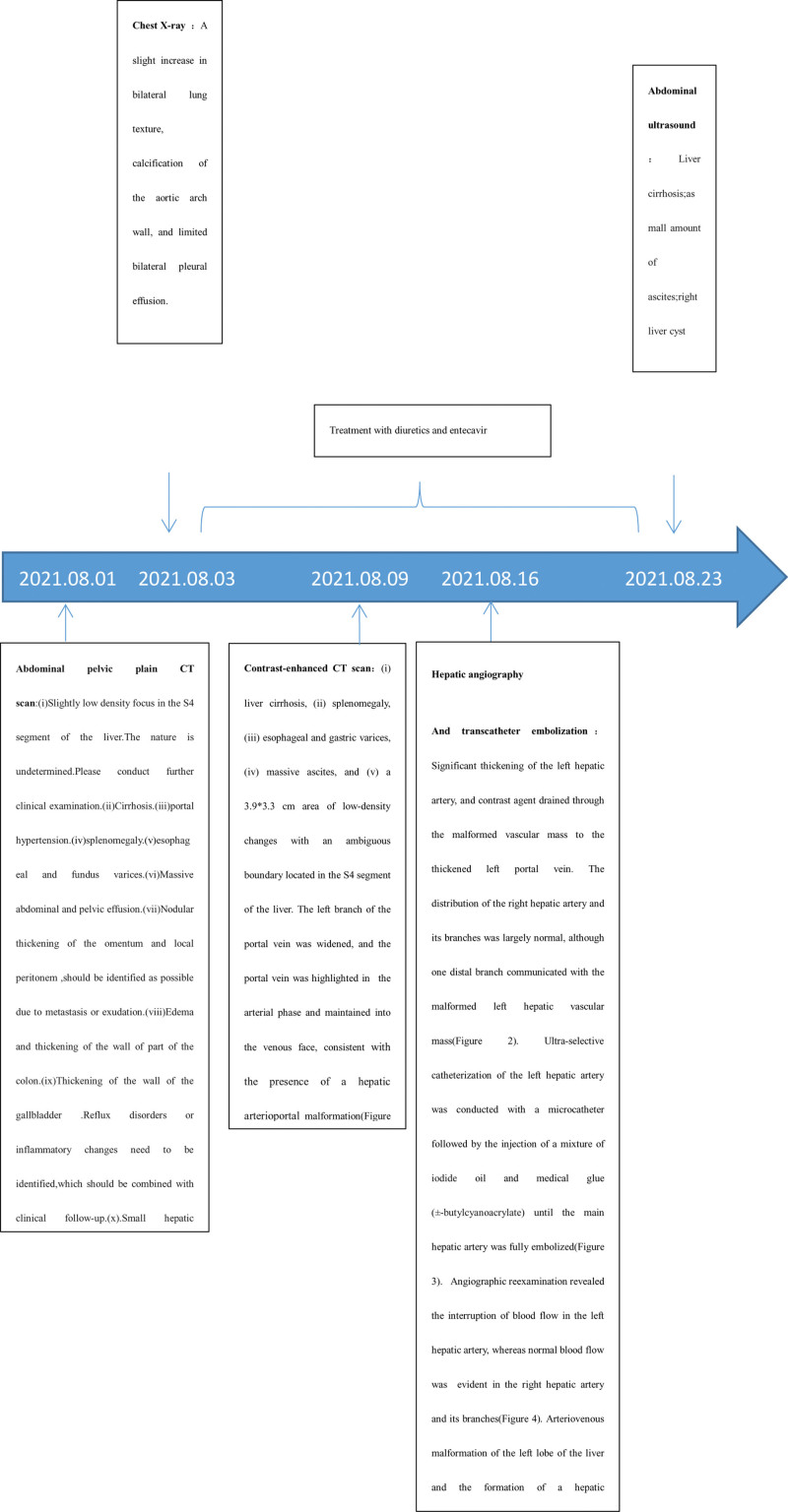
Clinical timeline.

Postoperatively, diuretic efficacy (Spironolactone 20 mg po tid, Furosemide 20 mg by Micro infusion pump 0.33 mg/min qd) was better than that before treatment and the patient exhibited significant weight loss, with abdominal ultrasound reexamination revealing only a small amount of ascites (Fig. 5). The patient symptom of bloating were reduced and appetite improved. Examination results revealed abdominal distension disappeared, abdominal wall tension decreased and shifting dullness negative. And the patient had no obvious discomfort after operation and no postoperative complications.

## 3. Discussion

Arteriovenous malformations of the liver are rare, and a range of factors can contribute to arteriovenous fistula formulation, including both tumorous and non-tumorous factors. Tumorous causes can include HCC, bile duct carcinoma, hemangioma, and a range of rare cancers, whereas non-tumorous causes can include congenital birth defects, cirrhosis, trauma, infection, iatrogenic injury, and Budd-Chiari syndrome.^[3]^ These malformations can include hepatic arterio-hepatic vein and hepatic arterio-portal vein malformations, with each exhibiting distinct manifestations. Arterio-hepatic vein malformations generally cause symptoms consistent with congestive heart failure or other forms of heart disease, whereas arterio-portal vein malformations often present with symptoms of portal hypertension. On the whole, this disease presents similarly to hereditary hemorrhagic telangiectasia (HHT) of the liver (Osler-Weber-Rendu disease), which is an autosomal dominant vascular disease characterized by telangiectasia, arteriovenous fistula (hepatic arterio-portal vein and/or hepatic vein) formation, a lack of stretch fibers and smooth muscle in aneurysmal small vessels, capillary, arteriole, and venule wall thinning, and a single endothelial cell layer, with a high risk of tortuous dilation, rupture, and bleeding. It can occur in any part of the body, but most often presents as cutaneous mucus membrane telangiectasia or in the liver as a diffuse arteriovenous malformation. HHT is diagnosed based on the following: Spontaneous and recurrent epistaxis. Telangiectasias at characteristic sites such as the fingers, lips, or oral/nasal mucosa, Visceral arteriovenous malformations or telangiectasias, and a first degree relative with HHT (inheritance is usually autosomal dominant). Patients are classified as follows: 3 to 4 criteria: definite HHT, 2 criteria: probable HHT, 0 to 1 criteria: HHT unlikely.^[4]^

The patient described in this case report suffered from a hepatic arterial-portal vein fistula but did not exhibit any characteristic surface telangiectasia and thus did not meet the criteria for HHT diagnosis. The patient also had no history of trauma, tumors, or liver puncture biopsy procedures, nor did she have any history of hepatic arteriovenous malformations. These facts, together with the fact that the patient was diagnosed with hepatitis B-associated liver cirrhosis, suggested that this patient had exhibited hepatic arterio-portal shunt development as a consequence of liver cirrhosis. Such cirrhosis can result from the persistent damage and necrotic death of hepatocytes together with the activation of fibroblasts, resulting in connective tissue deposition, stem cell nodule regeneration, lobular deformation, structural and blood flow abnormalities in the liver vasculature, including increases in the levels of resistance and pressure within the retrosinusoidal vein. Cirrhosis is a prominent driver of non-cancer-related hepatic arterio-portal shunt development, which can be detected in up to 13% of patients undergoing angiographic imaging.^[5]^ Nontumorous arterio-portal shunt development in liver cirrhosis patients is thought to be secondary to the occlusion of the small hepatic venules and the retrograde transsinusoidal filling of the small branches of the portal vein,^[6–8]^ which becomes a draining vein rather than one that supplies blood. These changes also coincide with compensatory increases in hepatic arterial flow that result in the establishment of a functional arterio-portal shunt.^[9]^ Prolonged shunting between the hepatic artery and portal vein can lead to the aggravation of cirrhosis. In addition to causing presinusoidal portal hypertension, artery-portal vein fistulae can aggravate extant portal hypertension in cirrhosis patients, potentially resulting in an increase in symptom severity and decompensation. This patient was affected by refractory ascites, highlighting a need for treatment. Following embolization, this patinet ascites rapidly diminished, supporting a major role for hepatic arterio-portal shunt development in the onset of refractory ascites in this patient.

A range of treatment strategies for hepatic arteriovenous shunt have been established to date, including hepatic artery embolization, the surgical ligation of the implicated hepatic artery, and partial hepatectomy. Of the possible interventions, liver transplantation is reserved as a final option, whereas interventional radiological treatment is generally the preferred treatment option. Embolization can decrease morbidity and shorten the duration of hospitalization for affected patients. The primary risks associated with the embolization procedure include the fact that the embolization material may migrate from the initial site into the portal system due to a high flow rate at the site of the established fistula, and that this embolization material may migrate to another unintended site. Only when embolization is unsuccessful is surgery indicated.^[10]^

In this case, the patient underwent transcatheter embolization for hepatic arteriovenous malformation, after which her ascites resolved with good short-term curative efficacy, although the assessment of long-term curative efficacy will require further observation. From this case, we learned that the patients who suffered from liver cirrhosis combined with hepatic artery-portal vein malformation and refractory ascites, active transcatheter embolization may get better therapeutic effect. However, it should be noted that this case is an individual case and cannot represent the situation of all patients. The practical data of a large number of clinical cases are still needed to confirm the effectiveness and safety of this treatment.

## Author contributions

**Data curation:** Zhenyu Ge, Kai Wang, Zhaomei Zhang, Peng Sun, Tingting Shen.

**Formal analysis:** Yang Tan.

**Investigation:** Hongsheng Dai.

**Methodology:** Ning Chen.

**Resources:** Xiaoqian Zhang.

**Writing – original draft:** Zhenyu Ge.

**Writing – review & editing:** Wenwen Li.
